# Chronic Recurrent Multifocal Osteomyelitis‐Like Paraneoplastic Syndrome as Initial Presentation of Nodular Sclerosis Classical Hodgkin Lymphoma

**DOI:** 10.1155/crh/9963997

**Published:** 2025-12-19

**Authors:** Michel Attieh, Seyed Reza Taha, Selim Nasser, Fouad Boulos

**Affiliations:** ^1^ Department of Anatomical Pathology and Laboratory Medicine, American University of Beirut Medical Center, Beirut, Lebanon, aubmc.org.lb; ^2^ Department of Pathology and Immunology, Washington University School of Medicine, St. Louis, Missouri, USA, wustl.edu; ^3^ Department of Pathology, Gilbert and Rose-Marie Chagoury School of Medicine, Lebanese American University, Byblos, Lebanon, lau.edu.lb; ^4^ Department of Pathology, Clemenceau Medical Center (Affiliated With Johns Hopkins International), Beirut, Lebanon

**Keywords:** Hodgkin lymphoma, nodular sclerosis classical Hodgkin lymphoma, paraneoplastic syndrome

## Abstract

Hodgkin lymphoma (HL) presenting with initial skeletal symptoms as a paraneoplastic phenomenon is extremely rare. Herein, we report the case of a 26‐year‐old man with nodular sclerosis classical HL (NSCHL) who presented with low back pain as the initial symptom. Imaging studies were unremarkable except for right S1–S2 sacral marrow edema on MRI, and multiple biopsies showed only inflammatory changes, resulting in a tentative diagnosis of chronic recurrent multifocal osteomyelitis. Later in the course of the disease, after performing routine series of MRIs, lymphadenopathy was finally detected. Core biopsies of the axillary and pelvic lymph nodes subsequently confirmed the diagnosis of HL. Complete resolution of bone lesions was observed following lymphoma treatment. This case highlights the diagnostic challenges of HL, particularly when it presents with rare skeletal paraneoplastic manifestations.

## 1. Introduction

Hodgkin lymphoma (HL) is a relatively rare lymphoproliferative disorder, accounting for approximately 10% of all new cases of lymphoma [1, 2]. HL is characterized by the presence of neoplastic Hodgkin Reed–Sternberg (HRS) cells in a background of non‐neoplastic lymphoid and inflammatory cells. HRS cells are derived from germinal center B cells that have undergone mutations, leading to the loss of most B‐cell‐specific markers and the aberrant expression of genes typically seen in other immune cell types [3]. The most common sites of disease in HL are the mediastinum and cervical lymph nodes, collectively accounting for 92% of cases [4]. Approximately 30% of patients with HL present with B symptoms, including fever, night sweats, and weight loss. However, most patients exhibit painless lymphadenopathy as the primary clinical manifestation [1]. HL is also known to manifest with various paraneoplastic phenomena, with neurological complications being the most common. However, nonmalignant multifocal bone involvement is a very rare paraneoplastic manifestation [5]. We discuss herein a case of nodular sclerosis classical HL (NSCHL), the most common subtype of classic HL, presenting with skeletal manifestations more than 1 year prior to diagnosis.

## 2. Case Presentation

The patient was a previously healthy 26‐year‐old male who began experiencing lower back pain radiating to the thighs. The pain initially occurred at night and in the morning but eventually became persistent throughout the day. The bone pain was also exacerbated by alcohol. It was responsive to single dose ibuprofen. A radiograph of the lower spine and pelvis was unremarkable. An MRI revealed edema at the level of S1‐S2, lateralized to the right, with a hypodense linear area on the T1 sequence. Both a stress fracture and an osteoid osteoma were considered as potential diagnoses. The patient was started on a regimen of etoricoxib 120 mg per day. The pain completely resolved for a week but then reemerged.

**Figure 1:**
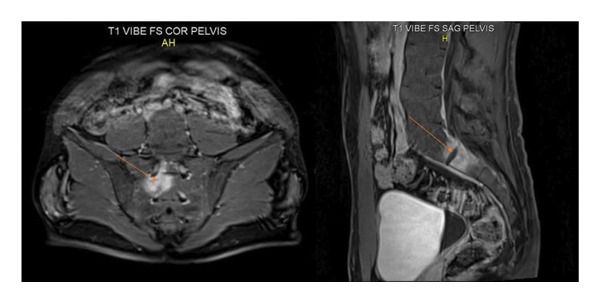
Dynamic MRI showing edema centered on the S1‐S2 disc, lateralized to the right of the midline, extending into the right S1 foramen.

A subsequent dynamic MRI revealed edema centered on the S1‐S2 disc, lateralized to the right side of the midline, and extending into the right S1 foramen (Figure 1). The radiologist’s interpretation favored an osteoid osteoma, though the diagnosis was not confirmed. The patient was started on a regimen of etoricoxib 60 mg per day and observed for 2 months, during which he experienced significant pain relief.

A follow‐up dynamic MRI was performed, showing a doubling of the edema; however, no soft tissue component was identified. A percutaneous biopsy of the affected region was performed to rule out primary bone lymphoma or osteoid osteoma. Pathology revealed dense intertrabecular fibrosis with chronic inflammatory cells, including lymphocytes and scattered eosinophils. CD3, CD5, CD20, CD30, and BCL6 immunostains demonstrated a mixed population of T‐cells and B‐cells, with no evidence of neoplasia.

**Figure 2:**
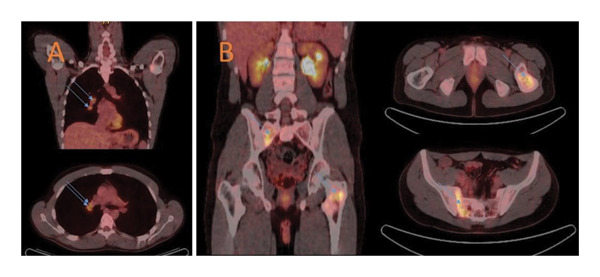
PET/CT scan showing abnormal activity in the sacral region, an abnormal signal in the left greater trochanter and adjacent femoral neck (SUVmax of 4), and two active right hilar lymph nodes measuring 1.5 cm each (SUVmax of 4.7).

A PET/CT scan was performed and showed abnormal activity in the same sacral region, and an abnormal signal associated with the left greater trochanter and adjacent femoral neck with an SUVmax of 4. In addition, there were two active right hilar lymph nodes measuring approximately 1.5 cm each with an SUVmax of 4.7 (Figure 2). Multiple percutaneous biopsies from both bone locations were performed, and pathology again demonstrated fibrosis with a chronic inflammatory infiltrate. Immunostains for cytokeratin, synaptophysin, CD3, CD5, CD10, CD20, and CD30 showed no evidence of a neoplasm and were more indicative of a nonspecific inflammatory process. The pathology samples were sent to a major referral center for consultation, and the results were similar to local interpretations.

Follow‐up MRI 2 months later revealed stable bone lesions and hilar lymph nodes, but a new lymph node measuring 1.2 cm was noted in the retroperitoneal paracaval region. A decision to perform an open biopsy of the left hip was made. The histology was similar to the previous ones, but additionally, it showed a proliferation of small blood vessels lined by plump epithelioid endothelial cells, in a background of prominent chronic lymphoplasmacytic and eosinophilic inflammation within a collagenous to myxoid stroma. The consensus diagnosis was consistent with epithelioid hemangioma/angiolymphoid hyperplasia with eosinophilia.

This was, however, disputed by expert consultants, who were in favor of an infectious process. A full infectious work‐up was, however, negative. Based on the multifocality of the skeletal lesions, and the negative infectious work‐up, a presumptive diagnosis of chronic recurrent multifocal osteomyelitis (CRMO) was rendered, and the patient was put on etoricoxib 90 mg a day with significant relief of symptoms.

Two MRIs over a series of 5 months showed an increase in axillary, pelvic, and para‐aortic lymphadenopathy, and progressive resolution of the sacral lesion. Although the lymph nodes were initially dismissed as reactive to the CRMO, their increased prominence led to a core biopsy. This revealed a population of lacunar Reed–Sternberg cells (positive for CD15 and CD30 and negative for CD3, CD20, and CD45) in a background of reactive lymphocytes with numerous eosinophils (Figure 3). The findings were diagnostic of NSCHL.

Figure 3(a) Hematoxylin and eosin stain of the lymph node biopsy with characteristic lacunar HRS cells. (b) CD30 immunohistochemical stain highlighting the HRS cells. (c) CD15 immunohistochemical stain highlighting the HRS cells.

**Figure:**
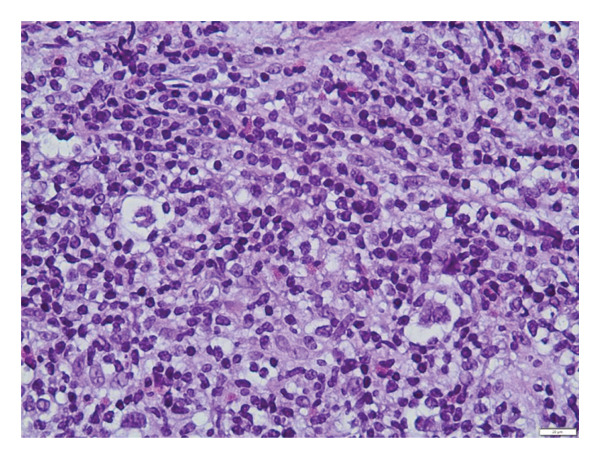
(a)

**Figure:**
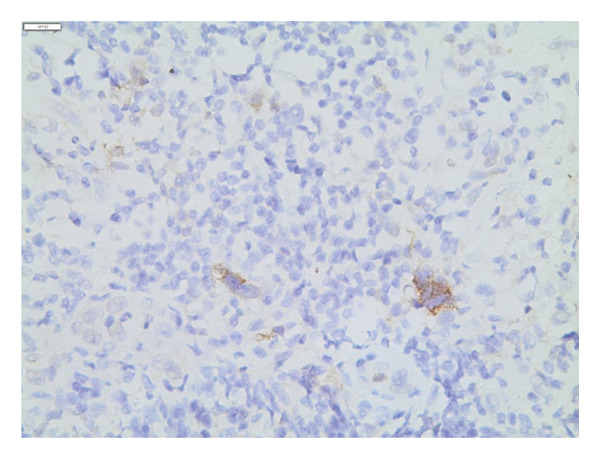
(b)

**Figure:**
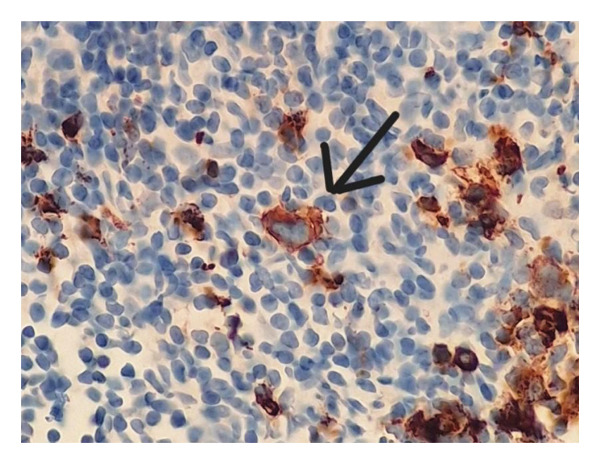
(c)

The patient underwent 6 cycles of chemotherapy with the adriamycin, bleomycin, vinblastine, and dacarbazine (ABVD) regimen. After the second cycle, both clinical symptoms and abnormal radiological findings were resolved. The patient remains in full remission 60 months postchemotherapy.

## 3. Discussion

NSCHL is the most common type of classic HL, primarily affecting young adults. Thanks to modern chemotherapeutic advancements, NSCHL now carries an excellent prognosis, with a cure rate exceeding 90% [6]. The hallmark of NSCHL is the presence of malignant HRS cells within nodules composed of extensive non‐neoplastic immune cells. A defining feature of NSCHL is the presence of lacunar cells, a subtype of HRS cells with abundant clear cytoplasm caused by shrinking artifacts. Immunohistochemically, HRS cells in NSCHL are typically positive for CD30 and usually positive for CD15, while lacking expression of CD45, CD20, and CD3 [6–8].

Occasionally, classic HL poses diagnostic challenges to pathologists and clinicians, as it can be mimicked by various other diseases, including T‐cell lymphomas and large B‐cell lymphomas [9–11]. Some reports have also shown that HL can rarely present with osseous involvement mimicking osteomyelitis, which can delay the diagnosis [12–15]. The complexity of diagnosis can also be compounded by extranodal paraneoplastic manifestations, masquerading as neurological, dermatological, hematological, and renal conditions [5]. However, osseous paraneoplastic manifestations of HL have rarely been described in the literature. One such case was reported by Pham et al., describing the occurrence of bone pain with negative biopsies and a working diagnosis of CRMO, which eventually culminated in a diagnosis of HL [16]. Another rare yet peculiar manifestation of HL is alcohol‐induced pain. Alcohol‐induced pain usually occurs in the vicinity of lymph nodes involved by HL, but it interestingly preceded the HL diagnosis in this patient and was part of his prodromal paraneoplastic symptomatology [17, 18].

Our case raises the possibility of a paraneoplastic inflammatory bone process associated with HL, although this cannot be established with certainty, particularly given the complete resolution of bone lesions following lymphoma treatment. Notably, the sacral marrow edema regressed on MRI while the patient was taking etoricoxib and before initiation of lymphoma‐directed therapy, whereas nodal disease progressed over the same interval. Although the possibility of bony involvement by HL cannot be entirely ruled out due to limited sampling and characteristic paucity of neoplastic cells, the absence of neoplastic HRS cells in multiple biopsies from two skeletal sites, including an open biopsy, argues against overt osseous involvement, although microscopic involvement cannot be excluded. Moreover, PET uptake at the bone sites was modest (SUVmax approximately 4), and there was no discrete soft‐tissue mass, findings supportive, though not specific, of inflammatory osteitis rather than overt osseous HL. The overlap between the paraneoplastic manifestations and a known rheumatologic disease, CRMO, further added to the diagnostic challenge. CRMO is characterized by an insidious onset of pain and localized tenderness over affected areas of bone. It is a diagnosis of exclusion, and patients should meet the so‐called Bristol criteria. Key diagnostic criteria include persistent bone pain, multifocal bone edema on imaging and mildly elevated inflammatory markers, such as a C‐reactive protein level typically below 30 mg/L [19]. The early radiologic improvement during etoricoxib therapy is consistent with a CRMO‐like inflammatory process. Given the cytokine‐rich inflammatory milieu integral to HL pathobiology, a paraneoplastic CRMO‐like mechanism remains biologically plausible, although unproven in this case [20].

The presence of these clinical and radiologic characteristics led clinicians to tentatively make a diagnosis of CRMO. Further complicating the case was the disagreement among pathologists regarding the interpretation of multiple bone biopsies in the search for a definitive diagnosis. At the treating institution, a diagnosis of epithelioid hemangioma of the bone was considered, while another major referral center favored an infectious process. However, neither diagnosis aligned well with the clinical nor expected findings of CRMO.

Later in the patient’s presentation, the associated lymphadenopathy was repeatedly dismissed as a reactive process to the bone lesions. However, as the sacral lesion resolved, the lymphadenopathy emerged as the key to the final diagnosis and appropriate management.

In conclusion, HL is a clinically diverse disease with multiple potential sites of involvement and possible paraneoplastic manifestations. The presence of persistent adenopathy in conjunction with other systemic manifestations should not be dismissed as a reactive process to the accompanying lesions. Clinicians should, therefore, have a low threshold for lymph node biopsy when systemic manifestations arise but lack a robust diagnosis based on the available clinical information and laboratory and radiographic studies.

## Consent

Written informed consent was obtained from the patient for the publication of this case and for the processing of personal data.

## Disclosure

All authors approved the final version of the manuscript.

## Conflicts of Interest

The authors declare no conflicts of interest.

## Author Contributions

Michel Attieh, Selim Nasser, and Seyed Reza Taha collected clinical data and drafted the manuscript. Michel Attieh, Seyed Reza Taha, and Fouad Boulos reviewed and edited the manuscript. Fouad Boulos and Selim Nasser provided supervision and final review.

## Funding

No funding was received for this research.

## Data Availability

The data supporting this case report are not publicly available to protect patient confidentiality but may be provided by the corresponding author upon reasonable request, subject to ethical approval.
